# The efficacy and safety of local infiltration analgesia vs femoral nerve block after anterior cruciate ligament reconstruction: A retrospective trial protocol

**DOI:** 10.1097/MD.0000000000023895

**Published:** 2021-01-22

**Authors:** Juan Chen, Xiaowei Wang

**Affiliations:** Department of Anesthesia, Handan Central Hospital, Hebei, China.

**Keywords:** anterior cruciate ligament reconstruction, femoral nerve block, local infiltrative analgesia, study protocol

## Abstract

**Background:**

Several previous trials have attempted to compare the efficacy of femoral nerve block (FNB) and local infiltrative analgesia (LIA) for patients received anterior cruciate ligament (ACL) reconstruction, but reached inconsistent conclusions. The primary purpose of this present research was to compare the FNB and LIA in the reconstruction of ACL.

**Methods:**

This investigation was conducted and then reported on the basis of Strengthening the Reporting of Observational studies in the Epidemiology checklist. From our registry database, we retrospectively determined 688 patients who received the primary reconstruction of ACL from 2016 to 2019 at our academic institutions. This current retrospective cohort study was approved through the institutional review committee at our hospital. Inclusion criteria contained the primary or autograft bone-patellar tendine-bone reconstruction of ACL in the patients over 16 years of age. Patients in the LIA group underwent intraoperative infiltration at the harvested site after tendon harvest, with use of 2 mg/mL of ropivacaine 20 mL and 5 mg/mL of epinephrine, respectively. After the reconstruction of ACL, 5 Lg/mL of epinephrine, and 20 mL of ropivacaine (2 mg/mL) were injected at the site of surgical trauma. The patient in FNB group was given 40 mL of ropivacaine (2 mg/mL), and the ropivacaine was injected into femoral nerve sheath at femoral triangle level. The primary outcome was the consumption of morphine 24 h after the operation. And the secondary results involved the complications, functional results, and the scores of pain.

**Results:**

It is assumed that the efficacy of LIA in the early postoperative pain is no less than that of FNB. For our study, the major limitation is the lack of randomization. Nevertheless, these data were prospectively harvested, with high response rate of patient.

**Trial registration:**

This study protocol was registered in Research Registry (researchregistry6277).

## 1 Introduction

The tears of anterior cruciate ligament (ACL) are one of the most prevalent orthopedic injuries in the United States, and the overall incidence rate is 68.8 cases per 100 thousand person per year, and the incidence rate is increasing year by year.^[1,2]^ As a kind of adjunct to the programs of conventional pain control, the use of regional anesthesia in ACL reconstruction (especially peripheral nerve block) to control the pain has become more and more common, because it can control local pain without affecting the whole body, thereby decreasing the need of general anesthesia, it may also reduce the opioids burden after the operation.^[3–6]^

Traditionally, the femoral nerve block (FNB) is considered to be the best peripheral nerve block for the reconstruction of ACL, due to it has been proved to reliably offer adequate analgesic effect in a lot of randomized experiments. Nevertheless, many investigations have confirmed the risk of the FNB nerve damage owing to direct trauma or neurotoxicity from injected anesthetics.^[7–10]^ Furthermore, it is also related to the quadriceps weakness after operation, which may lead to the prolonged recovery time and delayed limb activity. An alternative approach is local infiltrative analgesia (LIA), which was developed more than 40 years ago and involves injecting a local anesthetic into the orthopedic site.^[11,12]^ The simplicity of this technique and the accessibility of leg movements are two main reasons for the widespread use of LIA by orthopedic surgeons.^[13]^

Several previous trials have attempted to compare the efficacy of these 2 techniques for patients received ACL reconstruction, but reached inconsistent conclusions.^[14–16]^ Due to the limited sample size, these researches failed to draw a clear conclusion. Hence, the primary purpose of this present research was to compare the FNB and LIA in the reconstruction of ACL. It is assumed that the efficacy of LIA in the early postoperative pain is no less than that of FNB.

## 2 Materials and methods

### 2.1 Study design

This investigation was conducted and then reported on the basis of Strengthening the Reporting of Observational studies in the Epidemiology checklist. From our registry database, we retrospectively determined 688 patients who received the primary reconstruction of ACL from 2016 to 2019 at our academic institutions. This current retrospective cohort study was approved through the institutional review committee at Handan Central Hospital and then registered in the Research Registry (number: researchregistry6277).

### 2.2 Inclusion and exclusion criteria

Inclusion criteria contained the primary or autograft bone-patellar tendine-bone reconstruction of ACL in the patients over 16 years of age. While the criteria for exclusion contained age under 16 years, multiple ligament injury of the knee joint, allogeneic or autologous hamstring tendon transplantation, known intolerance or allergy to the bupivacaine or ropivacaine, and some other main concomitant operations, containing open surgery, meniscal transplantation, osteotomy, or the arthrotomy for any reason.

### 2.3 Procedural details

All the patients received arthroscopic reconstruction of ACL with autologous bone patellar tendon bone graft through the anatomic tunneling. Thigh tourniquet inflation was only utilized for the transplantation (first 12–15 min of surgery). The operation was finished through an experienced surgeon. In a typical manner, tibial tunnel was drilled through the incision in the graft collection area using the tibial drill guide, while femoral tunnel was drilled through anteromedial portal utilizing the overtop guide. All the patients were given general anesthesia in the process of procedure.

### 2.4 Anesthetic protocol

Patients in the LIA group underwent intraoperative infiltration at the harvested site after tendon harvest, with use of 2 mg/mL of ropivacaine 20 mL and 5 mg/mL of epinephrine, respectively. Along tendon extractor, a catheter was guided, and then analgesia was performed at the same time as covering incision to prevent the outflow of local anesthesia. After the reconstruction of ACL, 5 Lg/mL of epinephrine and 20 mL of ropivacaine (2 mg/mL) were injected at the site of surgical trauma. Intra-articular injection and the Infiltration were performed via a surgeon. The ultrasound guidance was utilized to properly locate the target nerve sheath and then the local infiltration was conducted in FNB group. In the process or after the operation, no nerve stimulators were utilized. The patient was given 40 mL of ropivacaine (2 mg/mL), and the ropivacaine was injected into femoral nerve sheath at femoral triangle level.

### 2.5 Outcome measures

The primary outcome was the consumption of morphine 24 h after the operation. And the secondary results involved the complications, functional results, and the scores of pain. The results related to pain contained the cumulative consumption of morphine at 2 and 48 h after surgery; the scores of dynamic and resting pain at 2, 24, and 48 h after surgery; and the incidence rate of nausea and vomiting at 2, 24, and 48 h after surgery. The functionally related results were range of motion, the distance of walking, and the quadriceps strength, all of these were detected at 24 and 48 h after surgery. At post-operative consultation, any complications associated with the surgery, for instance, the persistent hypoesthesia or novel sensory abnormalities, infection, hematoma, chondrolysis, the lower extremities weakness, or neuropathic pain, were sought.

### 2.6 Statistical analysis

The analysis of data was performed on the basis of intention to treat. The continuous variables are represented in terms of mean of 95% confidence intervals, ordinal variables in terms of quartile range and median, and the categorical variables in terms of the frequency. The comparison of non-parametric data and continuous parametric data were respectively conducted with Mann–Whitney *U* test and Student's *t* test. And the Pearson's test or the Fisher's exact test was used appropriately to compare the categorical and dichotomous data. Based on the two-tailed probability, significance was considered when *P* < .05. SPSS V22.0 software (IBM, Chicago, IL) was utilized to implement the statistical analysis.

## 3 Discussion

The reconstruction of ACL has been proved to be a cost-effective, effective, and safe method. Nevertheless, patients often experience moderate to severe pain after the surgery and need narcotic analgesia to control the pain, particularly in 24 to 48 h after the operation. Several randomised controlled trials have attempted to compare the efficacy of these 2 techniques for patients received ACL reconstruction, but reached inconsistent conclusions. Due to the limited sample size, these researches failed to draw a clear conclusion. Hence, the major target of this present research was to compare the FNB and LIA in the reconstruction of ACL. It is assumed that the efficacy of LIA in the early postoperative pain is no less than that of FNB. For our study, the major limitation is the lack of randomization. Nevertheless, these data were prospectively harvested, with high response rate of patient.

## Author contributions

**Conceptualization:** Xiaowei Wang.

**Formal analysis:** Juan Chen.

**Funding acquisition:** Xiaowei Wang.

**Investigation:** Juan Chen.

**Methodology:** Xiaowei Wang.

**Project administration:** Xiaowei Wang.

**Resources:** Xiaowei Wang.

**Software:** Juan Chen.

**Supervision:** Xiaowei Wang.

**Validation:** Juan Chen.

**Writing – original draft:** Juan Chen.

**Writing – review & editing:** Xiaowei Wang.
